# Interleukin-36 in Infectious and Inflammatory Skin Diseases

**DOI:** 10.3389/fimmu.2019.01162

**Published:** 2019-05-24

**Authors:** Anna-Lena Buhl, Joerg Wenzel

**Affiliations:** Department of Dermatology and Allergy, University Hospital of Bonn, Bonn, Germany

**Keywords:** IL-36, IL-36γ, inflammation, infection, skin, dermatosis, psoriasis

## Abstract

Interleukin-36 (IL-36) comprises to a cytokine family consisting of four isoforms IL-36α, IL-36β, IL-36γ, and IL-36 receptor antagonist (IL-36 Ra). These IL-36 cytokines, in turn, belong to the IL-1 superfamily. The IL-36 receptor (IL-1R6) is functional as a heterodimer formed of IL-1R6 and IL-1 receptor accessory protein (IL-1RAcP). IL-36α, IL-36β, and IL-36γ are regarded as pro-inflammatory ligands and IL-36 Ra as well as IL-38 as anti-inflammatory ligands of IL-1R6. IL-36 cytokines are mainly expressed on the barrier sites of the body e.g., bronchial, intestinal, and dermal epithelium. One of their most important biological functions is the bridging of innate and adaptive immune responses. A disturbed balance between pro-inflammatory and anti-inflammatory branches easily leads to inflammation of the corresponding tissue. The most prominent example for an altered IL-36 expression is the spectrum of psoriasis. In addition to inflammatory dermatoses, IL-36 also seems to play a role in infectious dermatoses. Microbial triggers, especially *Staphylococcus aureus* infection, increase the production of pro-inflammatory IL-36 cytokines and initiate/promote the inflammation of skin lesions. Due to the discovery of IL-36 as an important immune mediator, it has already been possible to develop important diagnostic tools for dermatitis. Not only in the field of inflammatory skin diseases, but also in pulmonary and intestinal inflammation, there is evidence that IL-36 cytokines might have diagnostic and/or therapeutic relevance.

## Introduction

Interleukin-36 (IL-36) is a member of the IL-1 superfamily discovered about 20 years ago (1–3). The four existing isoforms have been renamed several times (4). They were formerly known as IL-1F6, IL-1F8, IL-1F9, and IL-1F5. Since their functions were revealed about one decade ago, they were finally assigned as IL-36α, IL-36β, IL-36γ, and IL-36 receptor antagonist (Ra) (4, 5). While the isoforms IL-36α, IL-36β, and IL-36γ act as receptor agonists for pro-inflammatory functions (6), IL-36 Ra acts as an anti-inflammatory mediator (7). All IL-36 cytokines are encoded closely to each other on the human chromosome 2 within a cluster containing most of the remaining IL-1 cytokines (8–10). IL-36 cytokines are increasingly associated with inflammatory diseases. Among the associated diseases are Inflammatory Bowel Disease (IBD) (11, 12), rheumatoid and psoriatic arthritis (13), and various inflammatory and infectious skin disorders (14). Among the IL-36 associated skin diseases, psoriasis is the most prominent one (15–17), in which IL-36γ was identified as a specific biomarker (18). This review summarizes the current state of knowledge about IL-36, focusing on the special role of IL-36 in the dermatopathological context, and provides insights into the possibilities of using IL-36 as a potent pharmaceutical target in dermatology.

## Expression, Induction, and Regulation of IL-36

**Figure 1:**
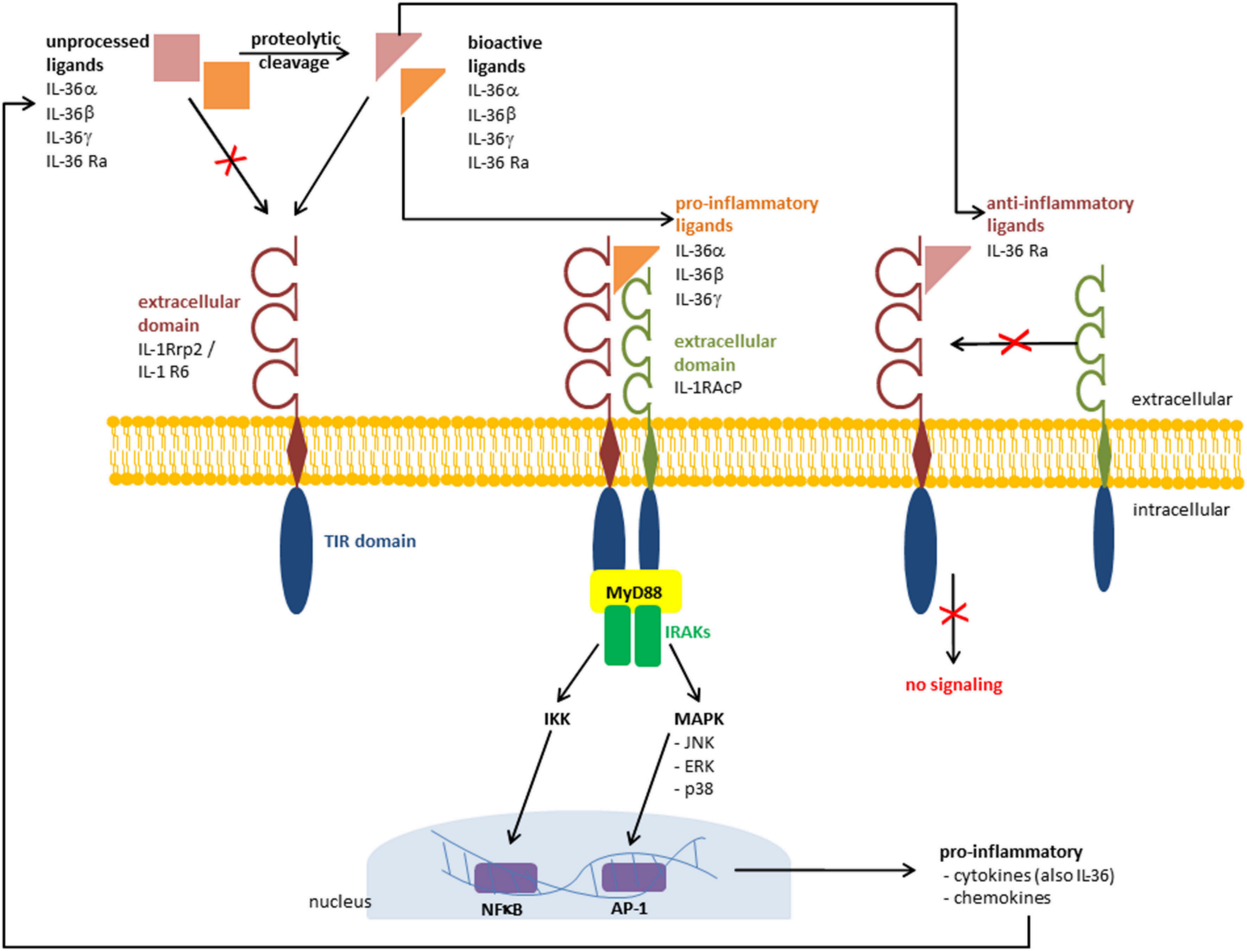
Receptor signaling pathway and recruitment of IL-1RAcP. Pathways that are activated by IL-36 cytokines via IL-36 receptor (IL 1Rrp2 /IL results in the γ, and IL 36β, IL 36α1R6). TThe binding of IL-36 activation of pro-inflammatory mediators. The binding of the anti-inflammatory ligand IL 36 Ra prevents the recruitment of IL 1 receptor accessory protein (IL-1RAcP) and the pro-inflammatory cascade is absent. TIR, Toll/Interleukin 1 receptor; MyD88, myeloid differentiation primary response 88; IRAKs, interleukin-1 receptor-associated kinases; NFB, nuclear factor “kappa-light-chain-enhancer” of activated B-cells; AP 1, activator protein 1.

The expression of IL-36 cytokines and IL-36 receptor (IL-1R6) has been described in many different tissues (Table 1). The underlying expression profile is more limited compared to that of the “traditional” IL-1 cytokines (19). IL-1R6 is mainly found on epithelial cells at the barrier sites of an organism (6, 15). The IL-36 isoforms IL-36α, IL-36β, IL-36γ, and IL-36 receptor antagonist (IL-36 Ra) are predominantly produced in the skin by keratinocytes (20). Furthermore, the isoforms IL-36α and IL-36γ are expressed in the respiratory tract (21) and IL-36β as well as IL-36γ are expressed in the intestines (22). The presence of IL-1R6 and the activity of the isoforms IL-36γ (23) and IL-36 Ra (24) was shown in murine glial cells, suggesting a role of IL-36 axis in brain physiology. It is also known that immune cells such as plasma cells, T-cells, macrophages and dendritic cells (DC) produce IL-36 under certain conditions (2, 20, 25), such as inflammation due to pathological changes. The expression of IL-36 cytokines and their regulation in the skin is well-studied. Some reports suggest a role of epidermal growth factor (EGF) in the regulation of IL-36α and IL-36β in the skin (26, 27). Furthermore, the transcription factor T-bet was found to regulate IL-36γ in myeloid cells (28). Takaishi et al. treated the skin of mice with imiquimod. Compared to wild type mice, there was a stronger, no self-limiting skin inflammation in immunoregulator Regnase 1 (Reg-1) knockout mice with higher IL-36α levels (29). This uncontrolled inflammation was attenuated in mice carrying a double knockout of Reg-1 and IL-1R6, suggesting a “brake”-like, regulatory function of Reg-1 in the IL-36/-IL1R6 signaling (29). Additionally, IL-36 Ra regulates IL-36 cytokine expression (Figure 1). It inhibits the pro-inflammatory cascade in an antagonistic pattern at the IL-1R6. The inhibitory IL-36 Ra was found to be upregulated in keratinocytes after stimulation with exogenous recombinant IL-36α, IL-36β, and IL-36γ (30). It is known that all IL-36 cytokines are produced and secreted in an inactive form without a caspase cleavage site. In contrast to the “traditional” IL-1 cytokines, IL-36 cytokines are regulated independently of the inflammasome (31). The production of IL-36 is induced by many triggers. Carrier et al. demonstrated the induction of IL-36 in keratinocytes by TNF, IL-17, IL-22, and IL-36 itself (16). This, in addition to the findings of Swindell et al. allows the assumption that IL-36 appears to be regulated by an autocrine feedback loop (Figure 1). In a monocytic cell line (THP-1) it was shown that the TLR2 and TLR4 ligands *Porphyromonas gingivalis* lipopolysaccharide (LPS) and *Escherichia coli* LPS led to an increased IL-36γ induction, but IL-36α and IL-36β were not altered (32). Furthermore, IL-36γ expression in normal human bronchial epithelial cells is increased after stimulation with the TLR3 ligand IL-17A (33). The induction of IL-36 expression by microbial stimuli together with the fact of predominant expression at barrier-sites (predominantly the skin), the IL-36 cytokine family is supposed to play an important role in terms of maintaining homeostasis and first-line defense mechanisms.

**Table 1:** Overview about IL-36 isoforms.

**Cytokine isoform**	**Former name**	**Tissue/cells capable of IL-36 expression**	**Activating proteases**	**Receptor signaling**	**Immune function**	**References**
IL-36α	IL-1F6	Skin Respiratory tract Bone-marrow Tonsils Lymph nodes Spleen Intestines Synovium (inflamed) Keratinocytes B-Lymphocytes Plasma cells T-Lymphocytes Dendritic cells Monocytes	Neutrophil derived proteases: Cathepsin G Elastase	IL1R6 with recruitment of IL-1RAcP	Pro-inflammatory	(2, 5–7, 11–13, 16, 19–21, 25–27, 31, 36, 49)
IL-36β	IL-1F8	Skin Respiratory tract Bone-marrow Tonsils Lymph nodes Intestines Neuron/Glial cells Heart Testis Synovium (inflamed) Keratinocytes B-Lymphocytes T-Lymphocytes Monocytes	Neutrophil derived protease: Cathepsin G	IL1R6 with recruitment of IL-1RAcP	Pro-inflammatory	(2, 3, 5–7, 16, 19–23, 25–27, 31, 36)
IL-36γ	IL-1F9	Skin Respiratory tract Intestines Brain Synovium (inflamed) Keratinocytes B-Lymphocytes Plasma cells T-Lymphocytes Dendritic cells Monocytes	Neutrophil derived proteases: Proteinase-3 Elastase	IL1R6 with recruitment of IL-1RAcP	Pro-inflammatory	(3, 5–7, 11, 12, 16, 19–22, 24–26, 31, 36, 49, 54)
IL-36Ra	IL-1F5	Skin Spleen Brain Heart Kidney Uterus Placenta Synovium (inflamed) Keratinocytes B-Lymphocytes Dendritic cells Monocytes Macrophages	Neutrophil derived protease: Elastase	IL1R6 w/o recruitment of IL-1RAcP	Anti-inflammatory	(1–3, 5–7, 9, 19, 21, 24, 31, 38)

## Receptor and Signaling Pathway of IL-36

The IL-36 receptor, also known as Interleukin-1 Receptor-Related Protein 2 (IL-1Rrp2) or Interleukin 1 Receptor Like 2 (IL1RL2) was finally assigned as IL-1R6 (34). Its ligands include all members of the IL-36 family: IL-36α, IL-36β, IL-36γ, and IL-36 Ra. Additionally, IL-38 is known to bind this receptor (35). The highest expression levels of IL-1 R6 can be found in skin and in mammary and mucosal epithelial cell lines (6, 7). The signaling pathway of IL-36 receptor agonists IL-36α, IL-36β, and IL-36γ is similar to that activated by IL-1α and IL-1β at the IL-1 receptor (7). The recruitment of IL-1 receptor accessory protein (IL-1RAcP) as a co-receptor induces the activation of nuclear factor-κB (NFκB) and activation of mitogen-activated protein kinases (MAPK) leading to the induction of pro-inflammatory cytokines, including (IL-12, IL-6, TNFα, and IL-23) (7, 31). Swindell et al. identified in IL-36γ-treated keratinocytes a large number of differentially regulated genes, including IL-1β, IL-36γ, and the NFκB-target genes TNFAIP3, NFKBIA, NFKB2, CXCL8, and BIRC3 (30). Interestingly, after silencing myeloid differentiation primary response gene 88 (MyD88) via CRISPS/Cas9, the IL-36γ induced alteration of gene expression was missing. This indicates that IL-36γ signaling is MyD88-dependent (30). In contrast to the pro-inflammatory receptor ligands IL-36α, IL-36β, and IL-36γ, binding of IL-36 Ra to IL-1R6 does not lead to the recruitment of IL-1RAcP (7, 31). Thus, the pro-inflammatory cascade is not switched on and the anti-inflammatory character of IL-36 Ra is achieved (Figure 1). IL-38 also binds to IL-1R6, and has similar anti-inflammatory effects to IL-36 Ra (35). However, it is not an IL-36 family member, but it also belongs to the IL-1 super family. van de Veerdonk et al. found that IL-36 Ra and IL-38 have nearly the same effects on immune cells. Both cytokines IL-36 Ra and IL-38 reduce the *Candida*-induced production of IL-17 and IL-2 (35). These findings indicate that not only IL-36 Ra but also IL-38 is a potent natural inhibitor of pro-inflammatory IL-36 cytokines.

## Processing and Secretion of IL-36

Comparable to IL-1α, IL-1β, and IL-18, all IL-36 cytokines, including IL-36 Ra are secreted in an inactive form. They are activated by different proteases upon N-terminal cleavage (31). Due to this cleavage there is an 500-fold increase in activity (36). The responsible proteases have been discovered already. The neutrophil-derived proteases cathepsin G and elastase activate IL-36α, cathepsin G activates IL-36β, whereas IL-36γ is activated by the proteases elastase and proteinase-3 (36). The proteases are able to process IL-36 either as free proteases, or as NET-bound proteases (37). In contrast to the IL-1 receptor antagonist, the antagonistic isoform of IL-36 also requires cleavage at the N-terminus for activation, which is performed by neutrophil elastase (38). It is known that IL-36α, IL-36β, and IL-36γ do not have a signal sequence or a caspase cleavage site, so they are assumed to be secreted by an alternative mechanism (2, 3, 31). However, regarding the secretion mechanism of IL-36, little is known so far. First studies in IL-36α overexpressing bone marrow-derived macrophages showed that IL-36α accumulates intracellularly and its secretion depends on LPS/ATP stimulus (39). Similarly, Lian et al. showed that stimulation of human keratinocytes with flagellin alone led to intracellular accumulation of IL-36γ and a release of IL-36γ was only detectable after stimulation with the RNA-analog polyinosinic-polycytidylic acid poly(I:C) (40). Based on these findings, Kovach et al. showed that IL-36γ is secreted by lung macrophages in an golgi-independent manner within vesicles and exosomes (41).

## Function of IL-36

The highest IL-36 activities probably are found at barrier sites of an organism (skin, lungs, and intestines). This indicates the importance of IL-36 cytokines in terms of protecting the body from the environment at its interfaces. The recognition of IL-36α, IL-36β, and IL-36γ by IL-1R6 leads to the activation of pro-inflammatory pathways resulting in a higher anti-microbial activity of corresponding cells. This includes an increased maturation/differentiation of murine and human myeloid cells (14, 25, 42–44), an increased bacterial clearance by murine macrophages in a sepsis-model (45), and an increased production of anti-microbial peptides by human keratinocytes (46). Furthermore, the production of pro-inflammatory mediators, such as cytokines and chemokines is induced by IL-36 receptor agonists in keratinocytes, Langerhans cells and macrophages. Among these mediators you can find cytokines, such as TNF, IL-6, and IL-8 (16, 47, 48), and chemokines, such as CXCL1, CXCL2, CXCL8, CCL3, CCL5, and CCL20. IL-36 signaling leads to the recruitment of leukocytes in human skin and lungs of mice (14, 49). Members of the IL-36 family are thought to have an important role in bridging the innate and adaptive immune systems. They do not only recruit and activate cells of the innate immune system, but they also have indirect and direct effects on the proliferation and plasticity of adaptive immune cells. It was shown, that IL-36 signaling appears to have a beneficial effect on T-cell proliferation (14). Furthermore, it helps polarizing naïve T-helper cells toward T-helper 1 cells in an IL-2 mediated fashion (50). Harusato et al. demonstrated that IL-36γ inhibits the differentiation of regulatory T-cells. IL-36γ promotes the T-cell polarization toward T-helper 9 cells leading to a strong T-cell driven inflammation of the intestine due to loss of self-tolerance (51). In contrast, other studies showed before a potential protective role of IL-36γ in acute intestinal inflammation. Mice lacking IL-1R6 showed a decreased resolution of intestinal damage after treatment with dextran sodium sulfate (22). The pro-inflammatory function of three agonistic IL-36 isoforms is opposed by IL-36 Ra, which has anti-inflammatory properties as a “natural inhibitor.” IL-36 Ra has several anti-inflammatory effects including the reduction of IL-17 and IL-22 production in peripheral blood mononuclear cells after *in vitro* Stimulation with *Candida* (35). Furthermore, the treatment of murine liver cells with recombinant IL-36 Ra reduces the production of chemokines, such as CCL20 and therefore prevents from proper tissue regeneration in acetaminophen-induced liver damage (52). Taken together, these findings indicate that homeostasis of many tissues is based on the intact balance between IL-36 receptor agonists and antagonists is essential for tissue homeostasis (53).

## IL-36 in Skin Diseases

As mentioned, an imbalance between the agonists and antagonists can lead to pathological changes. A dysregulation of both the pro-inflammatory IL-36 response and the anti-inflammatory IL-36 response can lead to damage of the corresponding tissue due to unnaturally strong inflammation or due to a lack of self-limiting features. An increasing number of infectious triggers of IL-36 production has been identified. Different *in vivo* and *in vitro* experiments showed that the bacterium *Pseudomonas aeruginosa* (54), and the fungus *Aspergillus fumigatus* (55) are able to induce IL-36 cytokines in lung tissue. Interestingly, viral infections such as chronic hepatitis B can lead to elevated IL-36 levels in blood serum (56). There is far more data available on many different pathogens and tissues (especially in the lungs) than shown here. However, in this review we focus on the role of IL-36α, IL-36β, IL-36γ, and IL-36 Ra in various infectious and inflammatory skin diseases.

### IL-36 in Infectious Skin Diseases

The family of IL-36 cytokines emerged from a common ancestor of IL-1 to escape resistance strategies of microorganisms against IL-1α, IL-1β and IL-18 (57). Thus, it is a valuable evolutionary advantage to have this cytokine family. This hypothesis is supported by the fact that IL-36 cytokines are highly preserved within many species. The skin has a special significance within the host defense against microorganisms. It is colonized by many microbes and skin cells as well as immune cells have to distinguish between commensals and potentially pathogenic microorganisms (58).

#### Bacterial Infections

One of the most prominent skin germ that regularly leads to infections is *Staphylococcus* (*S*.) *aureus*. These gram-positive cocci colonize the skin of about 10–20% of the healthy population as part of the normal flora (59). A significant proportion of all skin and soft tissue infections in hospital patients are caused by *S. aureus* infection (60). So far, research has mainly focused on subepidermal *S. aureus* models. The skin defense against *S. aureus* depends on the IL-1 receptor and MyD88 (61). Subsequently, two groups published their work on epidermal colonization by *S. aureus*, considering the interplay between *S. aureus* and keratinocytes (62, 63). According to Liu et al. IL-36α is predominantly produced upon superficial bacterial exposure, whereas IL-1β is produced after bacterial stimuli in deeper layers (63). Both groups describe phenol-soluble modulin α (PSMα) as the major virulence factor of *S. aureus* leading to the induction of IL-36α in keratinocytes. In addition, IL-36α induces an IL-17 mediated T-cell response promoting the inflammation of the skin. Colonization with *S. aureus* is particularly common in patients with atopic dermatitis (AD) (64). In most cases of a superinfected dermatitis there is a barrier defect in the skin, which enables the bacteria to invade. The order of events, whether the barrier defect of the skin is previously present or whether it is caused by bacteria, is still controversial. However, both events seem to favor each other and promote the inflammatory process. Antimicrobial therapies help to improve AD (65), which highlights the importance to consider superinfections of bacterial origins in dermatoses.

#### Fungal Infections

Similar to bacteria, there are also some fungal species that colonize humans as part of the skin flora and can lead to severe inflammations and diseases due to invasion (66). Also a fungal infection often occurs as a superinfection. For example, a large proportion of psoriasis patients additionally suffer from an infection with *Candida* species (67, 68). There is not much data concerning IL-36 expression in or by fungal infection of the skin, whereas psoriasis is a well-studied field of IL-36 research and is known for a high IL-36γ activity (18). Braegelmann et al. investigated for the first time the IL-36γ expression in the context of psoriasis and fungal infections. In addition to the histomorphological similarity between psoriasis and certain fungal infections, it was shown that the fungus species *Candida albicans* and *Trichophyton mentagrophytes* are able to induce IL-36γ (69). They concluded on the one hand that the inflammation of psoriatic skin might be driven by fungi and, on the other hand that the clinical picture of psoriasis might be caused by a misdirected IL-36γ reaction, which was originally directed against fungal infections. Furthermore, it was shown that oral epithelial cells of mice react with increased IL-36γ expression after *in vivo* stimulation with candidalysin (70). IL-36γ has a leading role in the defense against and clearance of fungal skin infections (69, 70).

#### Viral Infections

Many viruses, such as poxvirus, measles virus, and several viruses from herpes virus family are affecting the skin. First evidence for a role of IL-36 signaling in viral infections emerged when Kumar et al. demonstrated the induction of IL-36γ in keratinocytes by an *in vitro* herpes simplex virus (HSV) infection (3). Some years later it was shown that IL-36γ is induced in keratinocytes (40) and in vaginal/cervical epithelial cells (71) by the RNA-analog poly(I:C), simulating a viral infection, which supported the assumption of antiviral functions of IL-36 cytokines. Recent studies have further investigated the protective role of pro-inflammatory IL-36 cytokines in herpes infections. Herpes infections are very common in the population. About 90% of the population carries HSV-1 and about 20-25% HSV-2 (72). These viruses lead to an infection that persists for life and may have a very severe course under certain conditions. To date, there is no vaccination and no effective treatment available. Therefore, it is very important to understand the immunological processes of a herpes infection exactly. Milora et al. found increased levels of IL-36α mRNA and IL-36β mRNA in HSV-1 infected mouse skin (73). In human keratinocytes, *in vitro* stimulation with HSV-1 led to the induction of IL-36α, but not to the induction of IL-36β, and IL-36γ (73). In contrast, increased levels of IL-36γ were detected in vaginal epithelial cells upon HSV-2 infection (74). Subsequently, the application of exogenous IL-36γ in a three-dimensional human vaginal epithelial cell model was tested for its antiviral functions. It was found that exogenous IL-36γ is able to inhibit viral replication and induce a stable antiviral environment (74). Treatment with IL-36γ led to the production of pro-inflammatory cytokines, antimicrobial peptides and chemokines (e.g., CCL20) and was thus identified as a protective shield against HSV-2 (74).

### IL-36 in Inflammatory Skin Diseases

#### Psoriasis

**Figure 2:**
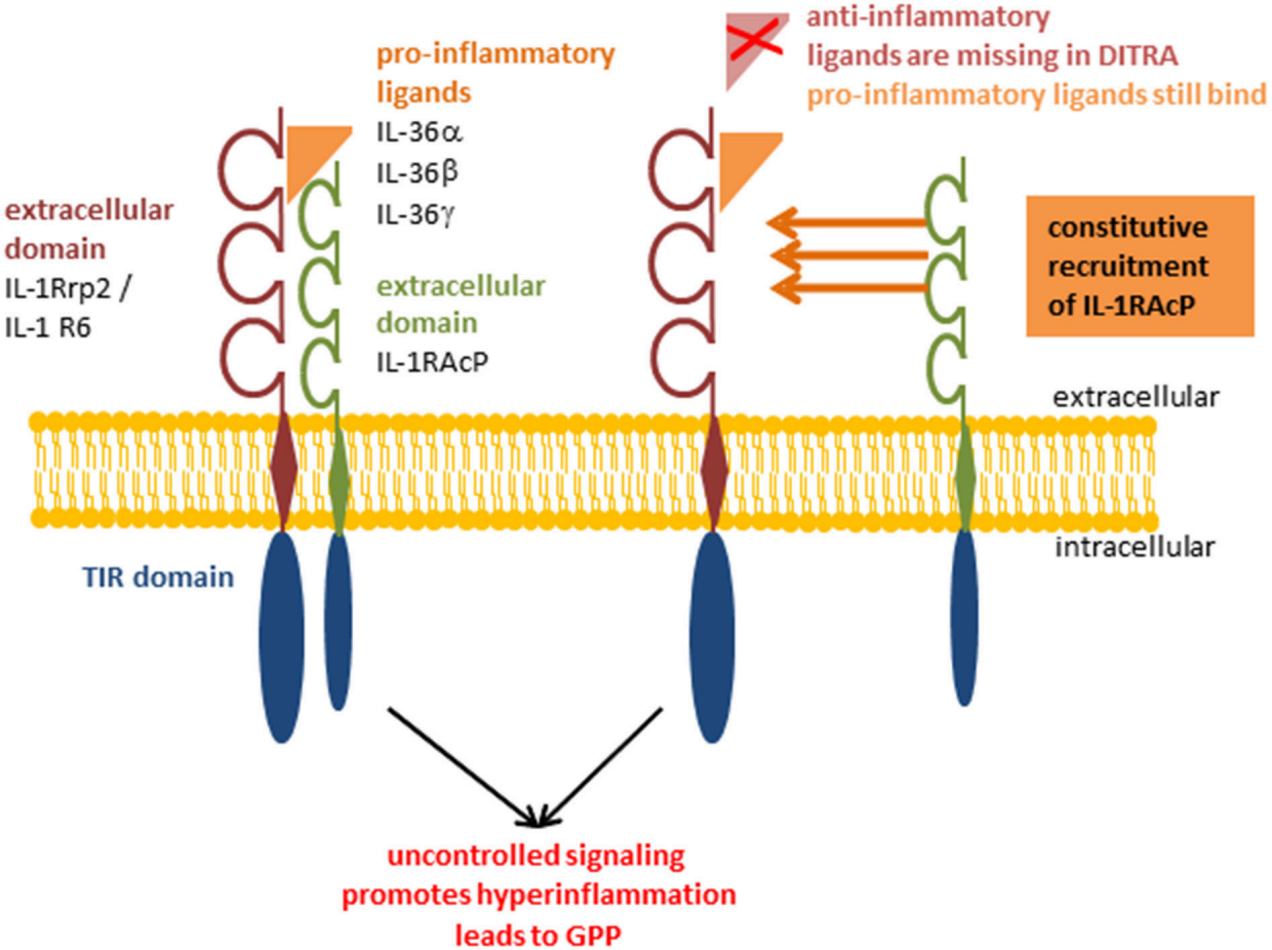
Deficiency of IL-36 Ra (DITRA). Pro-inflammatory IL-36α, IL-36β, and IL-36γ bind constitutively to the receptor inducing the signaling cascade without being regulated by anti-inflammatory IL-36 Receptor antagonist. Deficiency of IL-36 Ra (DITRA); generalized pustular psoriasis (GPP).

**Figure 3:**
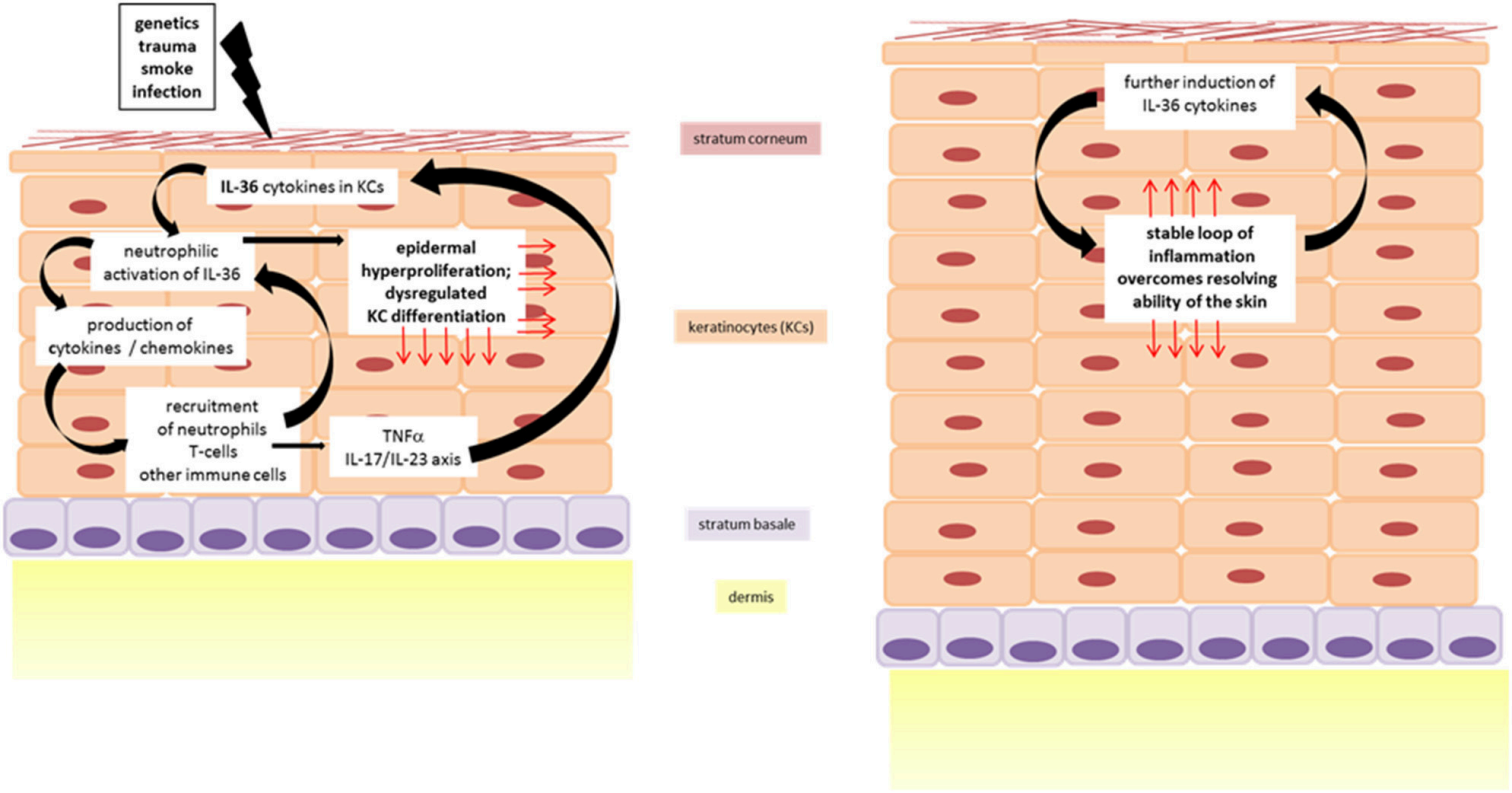
IL-36 driven inflammation of the skin in dermatoses. Cycle of inflammation is triggered by IL-36 driven skin inflammation leading to epidermal thickening.

Psoriasis is a chronic, relapsing, inflammatory disease of the skin. Even if the symptoms mainly affect the skin, they often also manifest in other parts of the body. Nail psoriasis causes nails changes and is thought to be a precursor of psoriatic arthritis which itself is manifested in the joints (75). Among psoriasis patients there is an increased risk of developing systemic comorbidities, such as cardiovascular disease (76) or metabolic syndrome (77). Although it is difficult to give correct information on epidemiology, it is often reported as 2% of the world's population (78). This difficulty could be explained by the fact that there is no clear clinical picture of psoriasis, but rather a spectrum of disease. Psoriasis palmoplantaris affects the palms of the hands and feet, psoriasis inversa the skin folds on the armpits and buttocks, and psoriasis capitis the scalp. The morphology of the skin alterations ranges from comparatively mild plaques, as they are found in the psoriasis vulgaris, over single, sterile, purulent blisters in pustular psoriasis, up to generalized pustular psoriasis (GPP), in which pus blisters occur extensively. GPP is known as the maximum and most severe variant of psoriasis and sometimes life-threatening. The disease of GPP is based on a missense mutation in the gene encoding for IL-36 Ra. Deficiency of IL-36 Ra (DITRA) results in a biochemically instable protein as well as disturbed receptor activity (Figure 2) (79–81). The pustular forms of psoriasis are characterized by a high genetic eruption pressure, which is why often small triggers (smoking, infections) are sufficient to elicit the disease. Since IL-38 was identified as another potent IL-1R6 antagonist, its role in psoriasis was currently investigated. IL-38 is downregulated in psoriasis patients and this correlates with disease severity (82). This study showed that both IL-38 and IL-36 Ra are able to reduce most of IL-36γ induced inflammatory processes *in vitro* in keratinocytes (82). Furthermore, pre-treatment with these antagonists a protective effect in imiquimod-induced psoriasis and saves mice from severe disease phenotype (82). Not only the loss of antagonistic activity, but also an increased agonistic activity at IL-1R6 is strongly associated with psoriasis. Gene expression analyses revealed an upregulation of all IL-36 family members (47, 83) but especially of the agonists IL-36α an IL-36γ (20). Interestingly, the isoform IL-36γ appears to play a specific role in psoriasis (18). The protein expression level of IL-36γ was up to three times higher in psoriatic lesions compared to other (inflammatory) skin disorders, such as atopic dermatitis (AD), lichen planus, contact eczema, pityriasis lichenoides, subacute cutaneous lupus erythematosus, and mycosis fungoides. The IL-36γ expression correlates with disease activity and decreases during TNFα treatment, which improves the disease (18). These findings indicate that IL-36γ is a potential biomarker for identification of psoriasis and monitoring of the disease course. It has been assumed for a long time that keratinocytes are the main modulators of psoriasis. However, it was found that a T-cell mediated immune reaction via the IL-17/IL-23/IL-22 axis including a significant importance of γδ T-cells contributes to the inflammation of the skin (84, 85). IL-36 cytokines are thought to be regulated by the IL-17/IL-23 axis during the course of psoriasis (16). Characteristics of psoriatic skin are an increased proliferation and impaired differentiation of keratinocytes. Pfaff et al. demonstrated within a three-dimensional skin equivalent that IL-36 cytokines are induced by IL-17 which results in the inhibition of keratinocyte differentiation. These findings indicate that an autocrine feedback loop between IL-36 cytokines and IL-17 contribute to the histological findings of epidermal thickening (Figure 3) (86). Furthermore, treatment with exogenous IL-36γ leads to a decreased expression of differentiation markers on keratinocytes (86, 87). In this context, Wang et al. identified the Wnt-signaling pathway as the responsible cascade for the altered differentiation and increased inflammation of keratinocytes in psoriasis (87). By the example of psoriasis, the importance of an intact balance between pro-inflammatory and anti-inflammatory processes becomes clear once more. Not only the deficiency of the receptor antagonist, as it is the case in GPP, leads to a disturbed balance. Also the hyper activation of IL-36 receptor agonists, such as an overexpression of IL-36α, which in transgenic mice led to psoriasis phenotype (15), interrupts the necessary homeostasis. There is an urgent need of understanding the underlying pathways. All these findings indicate that both IL-36 receptor agonists and antagonists represent potent therapeutic targets in the treatment of psoriasis patients.

#### IL-36 in Other Inflammatory Diseases

The prominent role of IL-36 in psoriasis raises the question whether IL-36 cytokines are of comparable importance in other inflammatory skin diseases. However, there are only few and partly contradictory data. A study whose cohort suffered from psoriasis and atopic dermatitis (AD) at the same time examined the intraindividual expression differences of psoriasis and AD lesions. Biopsies were taken from AD/psoriatic skin lesions and from non-lesional skin. As their RNA profiles were compared the IL-36 cytokine family was identified as “psoriasis-specific” (83). In contrast, another study reports an increased expression of IL-36α, IL-36γ, and IL-36 Ra in lesional skin of AD patients compared to non-lesional skin (88). Furthermore, an increased expression of all pro-inflammatory and anti-inflammatory IL-36 isoforms was demonstrated in the lesions of patients with allergic dermatitis by qPCR (89). Zebrowska et al. investigated on the involvement of IL-36 cytokines in the pathogenesis of some blistering diseases. They found significantly increased levels of IL-36α in correlation with a higher production of IL-17 in patients with bullous pemphigoid, pemphigus vulgaris, and dermatitis herpetiformis (90). Additionally, they describe a positive correlation between IL-36α and antibodies directed against transglutaminase which is the major autoantigen in dermatitis herpetiformis (90). With regard to inflammatory skin disorders the data is mostly limited to psoriasis. However, currently the discovery of new relationships is contributing to a better understanding of IL-36 itself and the associated diseases in order to treat or even prevent them.

## IL-36 as a Therapeutic Target

Inflammatory, non-infectious dermatoses are often treated non-specifically with anti-inflammatory agents such as corticosteroids. In the case of psoriasis, glucocorticosteroids and vitamin D3 derivatives are considered to be most effective. They are given either alone or in combination and either topically or systemically depending on the severity of the disease (91). These therapies were already used before molecular players of the disease were known. Recent studies show that these agents have an influence on the feedback loop between IL-36α or IL-36γ and the IL-23/IL-17 axis (92). It was shown in a murine psoriasis model that the vitamin D3 derivative calcipotriol inhibits the expression of IL-36α and IL-36γ in keratinocytes via their vitamin D receptor, which in turn prevents the infiltration of neutrophils (92) and saves skin from inflammation. Therapies used for more severe variants of dermatoses target the major pro-inflammatory cytokines, like IL-1 or TNFα and include immunomodulatory features (93, 94). The IL-36 cytokine family is part of the IL-1 superfamily. There are case reports in which patients suffering from GPP through DITRA have been successfully treated with Anakinra, a recombinant IL-1 receptor antagonist as a immunosuppressive agent (95–98). However, these cases were discussed as individual cases and the development of IL-36 specific therapies is requested (99). Systemic administration of any therapeutics can have severe side effects, which is why the research on even more specific, preferably non-systemic therapies is intensifying. It was shown that both specific (humanized) antibodies against the murine and human IL-1R6, as well as antagonistic substances of recombinant origin, lead to a reduced inflammatory response (100, 101). These results were proofed *in vitro* by determination of reduced production of IL-17 by keratinocytes and *in vivo* by determination of reduced skin thickening of mouse ears. Another therapeutic strategy aims to reduce the activity of the pro-inflammatory IL-36 receptor agonists by therapeutically inhibit the activating proteases cathepsin G and elastase. In contrast to former existing small-molecule inhibitors, which inactivate protease activities individually, Sullivan et al. identified bispecific peptide-based molecules, so-called “pseudosubstrates” that are able to antagonize the protease activity of both cathepsin G (usually would activate IL-36α and IL-36β) and elastase (usually would activate IL-36α and IL-36γ) simultaneously (102). A third strategy is the treatment with IL-36γ itself in terms of using its protective potential. It was shown in a mouse model that this treatment led to an antiviral environment. After infection HSV-2, the disease occurred significantly delayed with comparatively milder symptoms in mice which received IL-36γ (74). In this context, an increased and transient production of important immune mediators was observed, such as IL-36γ itself, IL-1β, IL-6, CCL20, CXCL1, and the antimicrobial peptide secretory leukocyte peptidase inhibitor (Slpi). The approaches aim to interrupt the IL-36 cascade in order to attenuate the inflammatory process. It is obvious that there is still a great potential to develop effective IL-36 related therapies and to conduct clinical studies on tolerability and efficiency. Life quality of dermatitis patients is diminished on one hand by the symptoms of the disease, but also frequently by social stigmatization. Therefore, there is a great need for therapy in the area of inflammatory dermatoses, whereby the cytokine family IL-36 seems to be an attractive target.

## Conclusion and Future Issues

IL-36 signaling is similarly to other members of IL-1 cytokine superfamily an effective first line defense mechanism. In contrast to the more general occurrence of IL-1 activity, the IL-36 associated immune response mainly takes place at the interfaces of an organism (intestines, lungs and skin). IL-36 emerges as the “optimized” version of a common ancestor and protects the organism at the corresponding sites against invasion of undesirable or even dangerous microorganisms. IL-36 cytokines regulate themselves by its natural antagonists, IL-36 Ra and IL-38, which prevents from hyper inflammation of the corresponding tissue in a healthy state. Signaling through IL-1R6 activates cascades including prominent pro-inflammatory transcription factors resulting in the production of pro-inflammatory cytokines, chemokines, and antimicrobial peptides. Finally, an inflammation is induced aiming the clearance of infections. As in any system with many players, a lot can go wrong. This potentially results in a disease. The responsible triggers and the molecular basis of existing forms of infectious and/or inflammatory dermatitis are highly variable and are not fully understood yet. It is striking that the different isoforms IL-36α, IL-36β, and IL-36γ are expressed differently under physiological as well as pathological conditions, although they basically have the same function. IL-36, with all its isoforms, is of great importance for the therapy of various dermatoses and also serves as a diagnostic tool in dermatology. It is very important to understand that IL-36 is bridging the innate and adaptive immune systems. Antagonizing the pro-inflammatory IL-36 cytokines was experimentally shown to be an effective treatment of dermatitis, like psoriasis. However, it is advisable to not only consider antagonizing the pro-inflammatory isoforms in order to reduce hyper inflammations but also use their protective potential. Recombinant IL-36α, IL-36β, and IL-36γ are potentially able to make barrier sites more resistant to the invasion of unwanted microorganisms. However, possible side effects of both approaches must be tested. We are at an early stage of research on treatment strategies involving IL-36 cytokines. There is still much to learn about this exciting cytokine family. There is a lot of diagnostic and therapeutic potential to be exploited, which could bring relief to many patients affected by dermatoses.

## Author Contributions

All authors listed have made a substantial, direct and intellectual contribution to the work, and approved it for publication.

### Conflict of Interest Statement

The authors declare that the research was conducted in the absence of any commercial or financial relationships that could be construed as a potential conflict of interest.
